# Photophysical Mechanisms of Photobiomodulation Therapy as Precision Medicine

**DOI:** 10.3390/biomedicines11020237

**Published:** 2023-01-17

**Authors:** Ann Liebert, William Capon, Vincent Pang, Damien Vila, Brian Bicknell, Craig McLachlan, Hosen Kiat

**Affiliations:** 1 Faculty of Medicine and Health, University of Sydney, Sydney 2006, Australia; 2 Adventist Hospital Group, Wahroonga 2076, Australia; 3 NICM Health Research Institute, Western Sydney University, Westmead 2145, Australia; 4 Faculty of Medicine of Montpellier-Nîmes, University of Montpellier, 34090 Montpellier, France; 5 Faculty of Health, Torrens University, Adelaide 5000, Australia; 6 Cardiac Health Institute, Sydney 2121, Australia; 7 ANU College of Health and Medicine, Australian National University, Canberra 2600, Australia; 8 Faculty of Medicine, Health and Human Sciences, Macquarie University, Macquarie Park 2109, Australia

**Keywords:** photobiomodulation, photophysical, oscillations, resonant recognition model, mechanotransduction, precision medicine

## Abstract

Despite a significant focus on the photochemical and photoelectrical mechanisms underlying photobiomodulation (PBM), its complex functions are yet to be fully elucidated. To date, there has been limited attention to the photophysical aspects of PBM. One effect of photobiomodulation relates to the non-visual phototransduction pathway, which involves mechanotransduction and modulation to cytoskeletal structures, biophotonic signaling, and micro-oscillatory cellular interactions. Herein, we propose a number of mechanisms of PBM that do not depend on cytochrome c oxidase. These include the photophysical aspects of PBM and the interactions with biophotons and mechanotransductive processes. These hypotheses are contingent on the effect of light on ion channels and the cytoskeleton, the production of biophotons, and the properties of light and biological molecules. Specifically, the processes we review are supported by the resonant recognition model (RRM). This previous research demonstrated that protein micro-oscillations act as a signature of their function that can be activated by resonant wavelengths of light. We extend this work by exploring the local oscillatory interactions of proteins and light because they may affect global body circuits and could explain the observed effect of PBM on neuro-cortical electroencephalogram (EEG) oscillations. In particular, since dysrhythmic gamma oscillations are associated with neurodegenerative diseases and pain syndromes, including migraine with aura and fibromyalgia, we suggest that transcranial PBM should target diseases where patients are affected by impaired neural oscillations and aberrant brain wave patterns. This review also highlights examples of disorders potentially treatable with precise wavelengths of light by mimicking protein activity in other tissues, such as the liver, with, for example, Crigler-Najjar syndrome and conditions involving the dysregulation of the cytoskeleton. PBM as a novel therapeutic modality may thus behave as “precision medicine” for the treatment of various neurological diseases and other morbidities. The perspectives presented herein offer a new understanding of the photophysical effects of PBM, which is important when considering the relevance of PBM therapy (PBMt) in clinical applications, including the treatment of diseases and the optimization of health outcomes and performance.

## 1. Introduction

Photobiomodulation therapy (PBMt), formerly known as “low-level laser” or “light therapy”, is the use of non-thermal light to enhance tissue repair and reduce pain [1,2,3]. While light therapy in its various forms has a long history dating back to ancient times and includes the advocation of natural lighting by Florence Nightingale and the treatment of various skin conditions by Niels Finsen at the turn of the 20th century, modern PBMt had its beginnings in 1967 with Endre Mester’s use of laser light to serendipitously heal skin conditions and regrow hair in a cancer model in mice [4]. The term low-level laser therapy (LLLT) has evolved into PBMt and includes light emitting diode (LED) devices. It is now used as a therapy for many conditions, including pain, tissue repair, inflammation, and neurological disorders. Over the decades PBMt has gained increasing acceptance; however, the full mechanisms of PBMt are yet to be entirely understood [3]. The action of photons on cytochrome c oxidase has been widely accepted as the primary component for the mechanisms underlying PBMt [3]. However, although cellular effects observed at red wavelengths were believed to primarily involve cytochrome c oxidase [5], new evidence has revealed that PBMt at 660 nm can enhance cell proliferation without cytochrome c oxidase modulation [6]. This revelation prompts the exploration of new perspectives on photobiomodulation (PBM) mechanisms and cellular interactions with light that may explain global effects within the body. In particular, the non-visual photophysical mechanisms of PBMt that are underpinned by bioelectromagnetic radiation and protein oscillations need to be revisited. This may unlock potentially novel intervention targets independent of cytochrome c oxidase that may enhance the potential of precision medicine using PBMt.

Mechanisms of PBM beyond the cytochrome c oxidase mechanism, including increased adenosine triphosphate (ATP) and the dissociation of nitric oxide (NO) from cytochrome c oxidase [7], have been proposed and investigated [3]. These include light-sensitive ion channels such as transient receptor potential channels that respond to low power laser irradiation [8], the increased direct synthesis of intracellular ATP [9], the modulation of mitochondrial and cell membrane-induced reactive oxygen species (ROS) that activate nuclear transcription factors [6,10,11,12,13], direct and indirect changes to oxidative stress [3], retrograde mitochondrial signaling [14], the modulation of electron transport chain enzymes and mitochondrial complexes (including the upregulation of complexes IV, negative regulation of complex III, and no regulation of complex II [15]), and other effects on gene expression [16].

This paper aims to investigate some direct and indirect systemic effects of PBMt that cannot be explained by mechanisms involving cytochrome c oxidase and how targeting these photophysical mechanisms may be important in the pursuit of photobiomodulation therapy as precision medicine. The seminal papers that guided the authors’ thinking are presented in Table 1. Photophysical mechanisms can be somewhat arbitrarily divided into biophotonic effects, mechanotransduction, and photophysical effects involving the cytoskeleton and oscillations of other proteins. The perspectives presented here may offer a new understanding of the photophysical effects in PBMt, which is important when considering the relevance of PBMt in clinical applications for the treatment of diseases and optimization of health outcomes and performance. These mechanisms are especially important for neurogenic conditions that involve the disruption of cortical coherence and brain wave patterns (e.g., alpha, gamma, theta waves), including migraine headaches with cortical spreading depression [17], and central pain syndromes, including fibromyalgia [18], Parkinson’s disease (PD) [19], and Alzheimer’s disease (AD) [20]. Additionally, PBMt has therapeutic potentials for diseases involving ion channel disruption, known as channelopathies, which include cardiac disease with dysautonomia and various dysrhythmia [21]. Notably, some wavelengths used in PBMt have been shown to have no therapeutic effect [22]. Here, it is proposed that additional insights into PBMt mechanisms might be made from the consideration of the photophysical effects of light.

## 2. Biophotons and PBM

One form of electromagnetic (EM) radiation in the body is known as biophoton emission, which refers to photons of light emitted by a biological system [23,24,26,30,61]. Biophotons, often referred to as ultra-weak photon emissions (UPEs), can be detected and measured by several techniques [62,63]. These emissions can originate when ROS form [64] in the cell and, in neurons, can influence potassium channel activity and be formed as a by-product of membrane depolarization [65]. Biophotons can also be released following endogenous ROS mechanisms in the mitochondrial and cell membranes, especially neurons of the central and peripheral nervous systems.

An interesting source of endogenous biophotons are those detected during neutrophil burst events [66,67], whereby the occurrence of oxidative stress appears to be quantifiable to UPEs detected as a biological readout [66]. This provides the basis of a potential non-invasive detection method that can be observed in real-time [55]. Indeed, circulating neutrophils in the bloodstream may have the capacity to be targeted for biophoton detection via the measurement of oxidative stress [30]. This is noteworthy since studies have shown that PBMt-stimulated oxidative bursts from neutrophils, as a measure of their production of ROS, resulted in increased functional profiles translating to elevated fungicidal capacity [68]. The manipulation of oxidative stress, particularly those deriving from neutrophil activity, is an interesting concept since it is understood that one of the key effects of PBMt in cellular systems is the modulation of ROS [3,10,69].

Recent studies have focused on the possible EM aspect of axonal conduction, including the optical propagation of photons through myelinic waveguides [70]. Axonal activity utilizes energy generation and exchange in the same way as other cellular and biological processes in the body. Early studies on neuronal function detected heat and infrared radiation transfers between the nerve during an action potential [71,72]. Further, the presence of infrared and visible light wavelengths has recently been shown in a variety of tissues and nerve cells [34,73,74]. In addition, the idea that photon emissions may be able to carry cellular information via the propagation of EM radiation has also been postulated [70]. If present, its implications for diagnostic and therapeutic use are significant.

An example of the potential importance of biophoton signaling is in the neuronal axon and dendrites. This could constitute a form of neural code and communication [59] and can be apparent in the central and peripheral nervous systems during periods of sleep and wakefulness [60]. This has been flagged as an important part of a systems biology approach [38]. New evidence has come to light in the explanation of this phenomena in the nodes of Ranvier [75].

Research studies have reported axonal responses in the presence of infrared and visible light irradiation in human neurons [76]. There has also been growing evidence for the endogenous generation of detectable cellular EM fields, with investigations on multiple biological effects that are attributed to a broad spectrum of wavelengths [77]. Additionally, it has been observed that emission intensity positively correlates with an increase or change in physiological activity, particularly under stress. For example, the presence of reactive oxygen species has been associated with the upregulation of UPEs [66,74,77,78].

In 2009, although it was well-known that biophotons existed in plants and bacteria, their existence and potential role as a cellular communication component in neurons was undetermined. However, there is now strong evidence for biophotons transmitting information within the body [79]. Like action potentials, biophotons may be involved in cell-to-cell communication to facilitate downstream cellular processes [70,80]. The potential effects of EM radiation within the infrared or visible spectrum on the expression of the endogenous biophotons have been postulated during neural excitability and signaling, culminating in a potential EM theory of neural communication [59,75]. Specifically, one breakthrough study showed that stimulating spinal motor or sensory nerve roots with light caused an increase in biophoton emissions at the end of the nerve root [34]. However, administering a neural conduction inhibitor blocked these effects. This result implies that biophotons are transmitted by axons in the same way as electrical signaling and are therefore likely to be a form of signaling [34]. 

**Figure 1:**
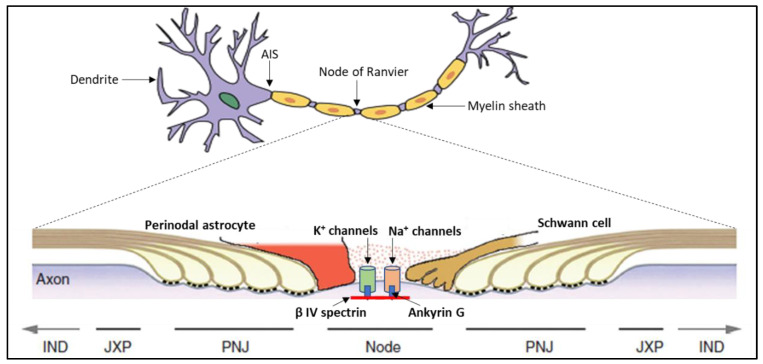
A detailed schematic of a Node of Ranvier.

Subsequently, our group had hypothesized that protein-to-protein interactions can occur when external photons are applied and proposed a mechanism of protein conformation to explain some of the effects of PBMt. A biophoton emission mechanism was hypothesized to be facilitated by these interactions, stimulated by the formation of ROS [38]. Following this hypothesis, a potential photophysical pathway has started to emerge highlighting the EM properties of neuronal axons and the emission of photons [75], particularly in the light-sensitive structures found in the gaps within the axonal myelin sheath called the nodes of Ranvier [81,82,83] (Figure 1).

The movement of propagated electrical signal between these nodes is significantly faster compared to signal transduction in unmyelinated axons, using a process called “saltatory conduction” [84]. Recently, studies investigating the EM properties pertaining to the physiology and function of the node of Ranvier have been conducted in both heart tissue and neurons [75,85], whereby EM-driven changes appear to affect a range of physiological pathways. For example, a recent study on EM radiation found that the accumulation of ion channels clustered in the nodes of Ranvier behaved like an array of nanoantennae emitting at wavelengths below 1600 nm [56,75]. This infrared emission is likely to propagate from node-to-node along the myelinated axon and may have an important role in nerve cell communication. It is possible that these EM emissions can trigger biological processes, such as triggering the release of neurotransmitters at the synaptic cleft, and may play an intimate role in neurotransmission [75].

The application of additional irradiation from an external source, such as through the delivery of PBMt, may be hypothesized to modulate the EM properties of these ion channels to elicit a biological response that has a direct influence on neurotransmission. Indeed, Chow et al. and others have shown that the application of low-level laser irradiation on neurons in-vitro, induced axonal varicosities in the same way as pharmacological anesthetics, resulting in the blockade of neurotransmission and therefore conferring an analgesic effect [32,86,87]. Interestingly, a recent study has suggested that neuronal spheroids may be involved in the pathology of Alzheimer’s disease [88]. From the model proposed by Zangari et al. [75], it is possible that the application of PBMt may act directly on the nodes of Ranvier and therefore modulate the signaling properties of proteins clustered within them, such as ion channels. This mechanism may be present in other cell types with similar light-responsive ion channels and has possible clinical implications for diseases involving channelopathies [89].

**Figure 2:**
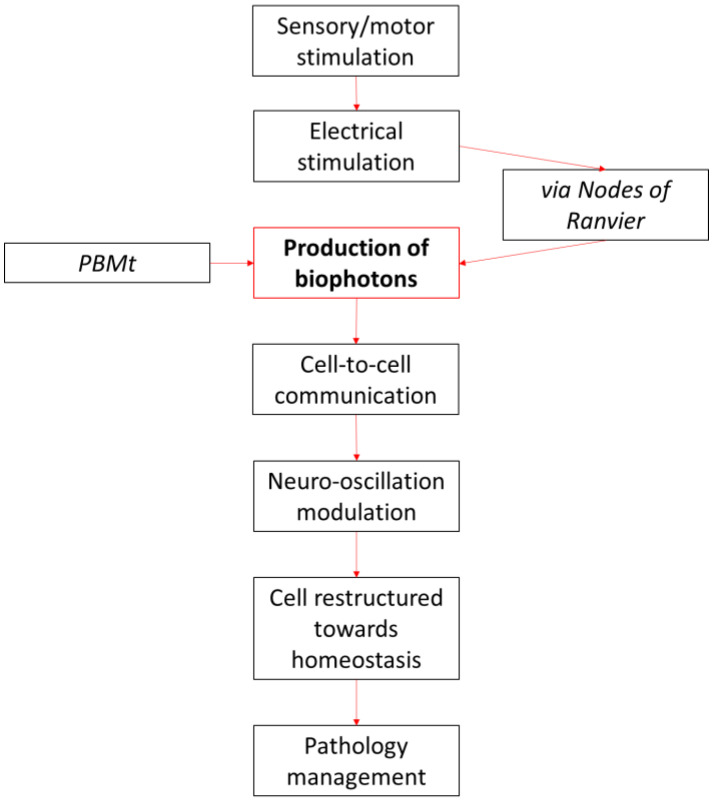
A conceptualization of how biophotons and photobiomodulation may contribute to cell-to-cell communication and the modulation of neural oscillations.

There is increasing interest in the connection between PBM and biophoton release [67]. The hypothetical connection between the production of biophotons and PBMt is shown in Figure 2. Placing intervention, biological target, and consequential downstream metabolite together provides potentially new hypotheses regarding the control and regulation of ROS in health, such as adaptive immunity and inflammation mechanisms from the production of nicotinamide adenine dinucleotide phosphate (NADPH)-derived ROS from phagocytes [90]. In this model, a lack of phagocytic ROS production may result in immunodeficiency and autoinflammation during an immune response; however, the overproduction of ROS can result in tissue damage and disease states due to cumulative oxidative stress [90]. The reports that biophotons influence aging [39] and metabolic disease states such as cardiovascular disease [91] and infectious disease [92] suggest that endogenous biophotons are not only present in physiological conditions but can also be used to quantifiably detect physiological events relevant to pathophysiology such as occurrence of oxidative stress. Therefore, they can also function as a potential target for the modulation of downstream effects. In summary, the neuroimmune modulatory effect of PBMt on production of biophotons through neutrophil burst may be an important aspect of the PBMt mechanism that has been so far under-researched due to previously unreliable technology to accurately measure biophotons.

## 3. PBMt and Mechanotransduction

**Figure 3:**
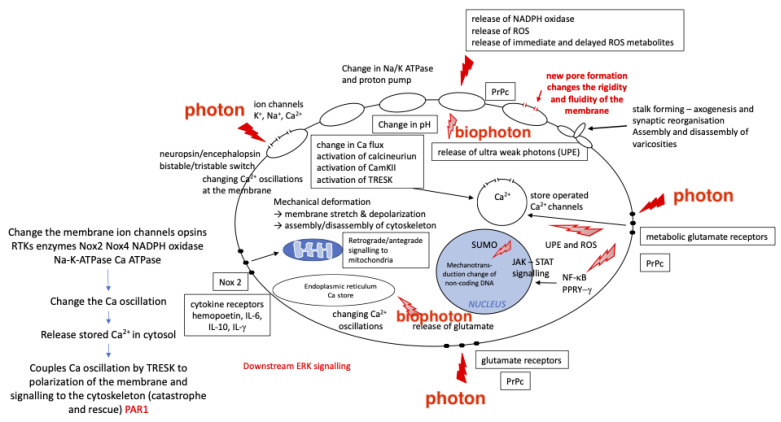
Mechanotransduction influences at a cellular level.

**Figure 4:**
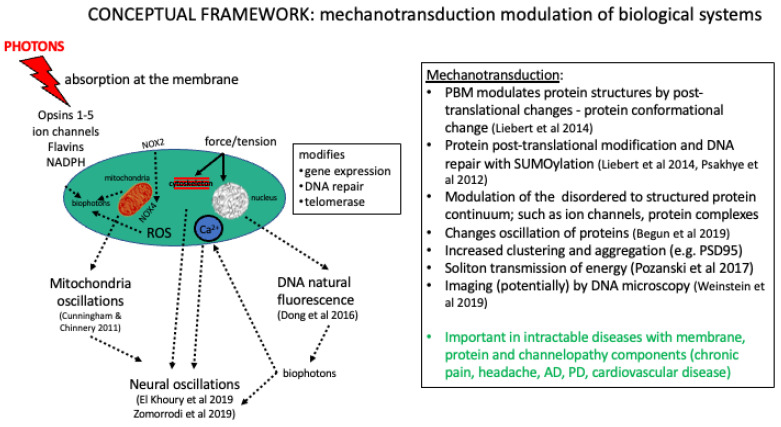
A conceptual framework for mechanotransduction effects of photobiomodulation [38,44,50,99,100,101,102,103].

**Figure 5:**
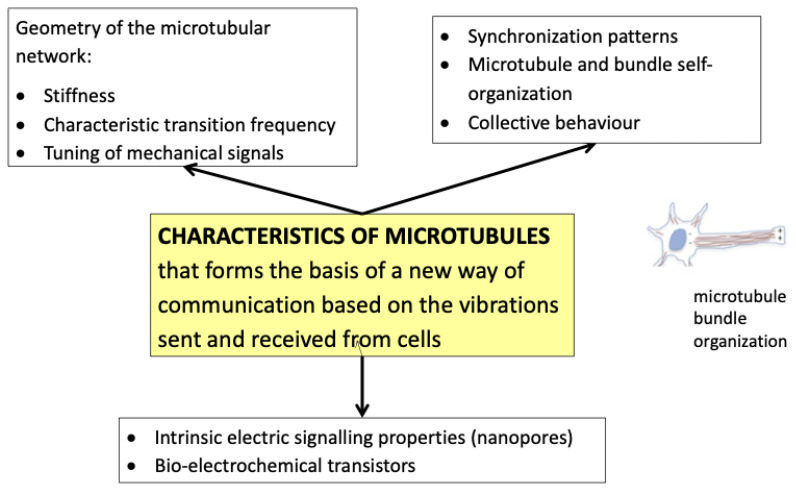
Proposed mechanism for cytoskeleton affecting global brain wave oscillation (adapted from Facchin et al., 2019 [49]).

The therapeutic effects of PBMt for related conditions including acute trauma from concussions and other traumatic brain injuries, degenerative diseases such as dementia, and behavioral and psychiatric disorders such as post-traumatic stress disorder, can be explained using the concept of biophysical alterations to the cell. These may include alterations to the cytoskeleton, the Ca^2+^ stores of the cell membrane, and oscillations of cellular proteins (see Figure 3). It is possible that PBM-induced changes in neuronal patterns and oscillations [50,93,94] are due to direct modifications of the cytoskeletal [45,95] (see Figure 4 and Figure 5). These may alter neurological electrical fields, associated brain wave pattern changes, subsequent symptomatic improvement, or possibly mitochondrial or Ca^2+^ oscillations [96]. Cantero et al. [45] have postulated that microtubules within neurons are able to generate electrical oscillations, modulate ion channels, and generate cytoskeleton regulated electrical activity (Figure 5). This may have implications for higher brain functions such as memory and consciousness. For example, improvements have been observed in PD patients treated with transcranial PBMt, with enhancements in mobility, balance, fine motor skills, and cognition [97]. Here, it is hypothesized that microtubule depolymerization and the accumulation of dopamine and ROS in the dopaminergic neuronal cytosol causes disruptions to the microtubule and cytoskeletal dynamic, which results in mitochondrial dysfunction and an increased risk of PD [98]. The application of transcranial PBMt to PD patients may augment dopaminergic neuronal microtubule bundles and enhance cytoskeletal function, thereby reducing microtubule depolymerization, ROS, and dopamine cytosol accumulation. This may reduce mitochondrial dysfunction.

### 3.1. PBMt Modulation of the Cytoskeleton

The cytoskeleton of the cell is made up of microfilaments, intermediate filaments, and microtubules. In addition to their biological functions, they have the capability to interact with electric signals, mechanical action, and EM fields to enable interplay between these properties. Mechanotransduction in cells (transduction of mechanical stimuli into biological signals) can be initiated when the microtubular network is exposed to EM fields in the THz frequency [95]. This means that light, as used in PBMt, is also able to modulate mechanotransduction in this system. Indeed, this mechanism may be involved in the reported effects of PBMt in cellular processes, including ATP synthesis [104], stem cell production [104,105], activation of ion channels (e.g., transient receptor potential (TRP) channels [106,107] and transient receptor potential cation channel subfamily V (TRPV) [108]), and reversal of neurological pathologies [109,110] such as in AD [111] and PD [112].

Expanding on this hypothesis, PBM has been demonstrated to induce observable changes to neuronal structures and mechanotransductive properties in the cytoskeleton, which seems to be a direct consequence of cytoskeletal modulation. Laser, but not LED, irradiation to the mouse dorsal root ganglion (DRG) neurons and cultured neonatal rat DRG neurons, using 830 nm and 20 mW continuous wave laser at doses of either 6 J over 5-min or 15 J for 15-min, resulted in “beading” or varicosity formation along the length of the neurons in the peripheral nervous system [32]. Additionally, live imaging using confocal microscopy showed the cessation of mitochondria movement along the cytoskeleton and accumulating at these varicosities. The formation of these varicosities reflects a disruption to the microtubular and cytoskeletal structures. This coincides with the decline in mitochondrial membrane potential (MMP) energy states, which are a reflection of ATP depletion within the mitochondria. Importantly, these varicosities are reversed 24 h after the cessation of PBM stimuli [32]. Similar changes have now been observed in the central nervous system, with improvements in synaptic plasticity and alterations in the cytoskeleton, including dendrites, with PBM [95,113,114].

There are several possible explanations for the physical and functional changes in neuronal cytoskeletal structure that relate to the non-phototransduction mechanisms of PBM. The polymerization and depolymerization of microtubules are energy-intensive process, occurring at about 10-min intervals. The reduction of available ATP halts polymerization, which leads to the disruption of cytoskeletal integrity and therefore the transport of signaling molecules and functions requiring ATP. This change in protein conformation can also be seen following absorption of light energy [115]. For example, the direct application of PBM at 810 nm to microtubules in-vitro causes incremental structural disassembly, resulting in a reduction in the rate and total amount of tubulin polymerization [38]. These effects appear to be dependent on the overall concentration of tubulin since as the tubulin content increases PBM irradiation also increases tubulin polymperisation rates and total polymer mass [57]. It is worth noting that laser irradiation also produces ROS including NO and singlet oxygen, which can lead to the disruption of the cytoskeleton, as demonstrated by the formation of varicosities in DRG neurons in response to hydrogen peroxide (H_2_O_2_) [116]. This possibly provides both a direct and an indirect mechanism, resulting in neuronal cytoskeletal modification.

### 3.2. PBMt Modulation of Ion Channels

It is also known that PBMt can directly target and modulate light-sensitive ion channels and signaling proteins to directly regulate microtubule function. Notable examples include firstly the weakly inward rectifying K channel (TWIK)-related spinal cord potassium channels (TRESK), which are important in photophobia, including in migraine with aura [86], and secondly the chromophore neuropsin. Both have important roles in neuroplasticity and memory and also regulate microtubule-associated protein 2 (MAP2) [117]. More recently, it has been determined that α-synuclein, which can modulate actin and microtubule activity in the cytoskeleton is important in neuronal function [118,119], with roles in axonal transport and the formation of the short microtubule subtypes [120]. Indeed, PBM has been shown to suppress the over-expression of α-synuclein in animal models of PD [121], which may occur as result of regulation of microtubule function by targeting relevant light-sensitive ion channels resulting in the modulation of neuroplasticity. A recent study by Buendia et al. [95] reported evidence of improvements in synaptic plasticity in a mouse model of AD, whereby application of transcranial PBMt resulted in the significant elevation of long-term potentiation in the Schaffer collateral fibers involved in signaling pathways between the CA3 and CA1 pyramidal neuron regions [122], potentially pointing to changes to a-amino-3-hydroxy-5-methyl-4-isoxanzolepopionic acid (AMPA) receptors and small-conductance Ca^2+^-activated K^+^ channel (SK2) channel plasticity, which in turn has been shown to participate in synaptic changes when an activity-dependent decrease contributes to changes in long-term potentiation [95].

### 3.3. PBMt Modulation of the Microbiome

There is also evidence to show that microbial-derived metabolites can induce actin cytoskeletal rearrangements with beneficial outcomes. Common microbial metabolites in the form of short-chain fatty acids such as butyrate and propionate caused observable alterations to filamentous actin directionality, with increased tight junction expression and protection from lipopolysaccharide-induced tight-junction mis-localization [123]. Blood-brain-barrier integrity was also improved with modulation of mitochondrial network dynamics [123]. Based on what is understood about the interaction of PBM with the gut microbiome [124], it is plausible that PBM-induced changes in microbiome populations would have a consequential impact on the circulating concentrations of microbial-derived metabolites and therefore possible actin cytoskeletal rearrangement.

### 3.4. PBMt and Glymphatic Clearance

The concept of cells undergoing a phase transition and the shifting of resonance (see Figure 4 and Section 4 below) may be applied to cellular proteins, such as α-synuclein, which may have significant implications in the progression of PD pathophysiology [125]. α-synuclein has, as part of its function, an antimicrobial peptide characteristic and is important for the homeostatic metabolic function of the immune system [126]. The prionization of α-synuclein is evident in skin samples of PD patients [127], and this mechanism results in a missense of monomers of α-synuclein to aggregate as oligomers, resulting in a loss of cellular function [128]. The α-synuclein protein is present in the cerebrospinal fluid (CSF) [129] and in red blood cells [130] with physiological functions within the lymphatics and is crucial in effective lymphatic drainage and control of tissues within the CSF. The latter has been shown in mice with A53T PD, demonstrating perivascular aggregation of α-synuclein and consequentially impaired polarization of aquaporin (AQP)4 [131]. Here, cervical glymphatic ligation causes severe dysfunction in mice, with an accumulation of misfolded α-synuclein, glial activation, inflammation, dopaminergic loss, and motor deficits. Based on these observations, it is highly probable that impairment of the brain’s glymphatic system can result in increased incidence of neurovascular, neuroinflammatory and neurodegenerative diseases, including the increased production of CSF by the choroid plexus and extra-choroidal sources, arterial wall pulsatility, the entry of CSF into the brain parenchyma via AQP4 channels, the accumulation of CSF within the perivenous space, and the disrupted function of the meningeal and cervical lymphatic vessels [132].

It is hypothesized that laser mechanisms underlying the positive effects of PBMt treatment for PD models [133] may have photophysical aspects related to the glymphatic system [132]. PBMt may be able to influence AQP function, including AQP4, by decreasing expression of the protein [134,135]. Since AQP4 supports CSF distribution, which is under circadian control, it is an important target in the reduction of circadian differences in drainage in the lymph nodes [60,136]. By extension, it is further hypothesized that PBMt may target the expression of AQP4. This is important because reduced AQP4 diminishes the difference in day and night levels of glymphatic influx and drainage to the lymph nodes, which suggests temporal considerations for the application of PBM in this regard.

Evidence of PBM effects on lymphatic drainage function in the brain reveal numerous recent animal studies that support the novel application of PBM to the cranial and extracranial lymphatics to control diseases where CSF outflow abnormalities are present [137]. For example, application of PBM was shown to improve amyloid-β clearance from the brain with a reduction in the density of smaller plaques [138]. This was postulated to be due to direct PBM-triggered control on lymphatic pumping and contractility, with observations that PBM application at low fluencies caused a relaxation of the mesenteric lymphatics and a reduction in systolic and diastolic contraction amplitude resulting in vessel vasodilation. This increase in lymphatic endothelium permeability allows for increased transport of larger molecules via the lymphatic vessels [139]. Interestingly, the same study reported that PBM application at higher fluencies resulted in complete blockage of vessel contractility [139], providing the possibility of fine control of lymphatic drainage as required by modulation of PBM parameters during application.

Another study reported that PBM stimulated clearing function with the accumulation of experimental tracers in the deep cervical lymph nodes from the cisterna magna. In addition, PBM-induced dilation of the mesenteric lymphatic vessels were also observed and associated with a reduction in the resistance to lymph flow [140]. Furthermore, it was also shown that the PBM irradiation of immune cells, such as macrophages, resulted in the upregulation of migration from the lymphatic vessels to the surrounding tissues, alongside increased lymphatic permeability. It is thought that this is likely to occur due to a reduction in transendothelial electrical resistance integrity and the overall expression of junction proteins such as vascular endothelial (VE)-cadherin [140].

PBM-treated AD mouse models displayed increased amyloid-β protein level accumulation in the deep cervical lymph nodes, potentially indicating an increase in the efficiency for PBM-induced stimulation of amyloid-β clearance from the brain [141]. It is possible that this increase in clearance may be a result of improvements in blood oxygen saturation, which may lead to improved mitochondrial ATP production that can stimulate lymphatic contractility to promote increased drainage and clearing activities within the meningeal lymphatic system [132]. Further experiments by the same group reported PBM associated enhancements in neurobehavioral status in these animals as a result of altered blood-brain-barrier permeability and possibly transendothelial integrity [140]. Another study that investigated the lymphatic clearance of other cell types, such as red blood cells, found that transcranial PBM application following intraventricular hemorrhage improved cell evacuation from the ventricles and enhanced symptomatic outcomes [142]. In this study, red blood cells were found to be transported from the ventricles to the deep cervical lymph nodes more quickly than non-irradiated animals, and the rate of red blood cell elimination was found to be higher following PBM intervention [142]. Furthermore, these animals also appeared to show faster recovery from intraventricular hemorrhage following PBM intervention, with a significant reduction in mortality and reduced stress compared to non-treated animals [142].

It is worth noting that changes to permeability and endothelial function within the clearance and drainage systems of the lymphatics may, in part, be due to increases in circulating NO. Indeed, PBM has been shown to increase blood flow in both humans and animals [143,144], based on the proposition that PBM causes the disassociation of NO from cytochrome c oxidase [3]. In previous work, PBM irradiation has been shown to increase neuronal NO from activation of endothelial NO synthase resulting in vessel vasodilation [144].

## 4. PBMt and Photophysical Mechanisms

### 4.1. Cell-to-Cell Oscillation

Interestingly, some proteins and networks in cells are comparable to vibrating systems that interact with each other and with other EM fields and hence transduce EM energy into mechanical force and other biological processes [49], which is another form of signaling in the body. In other words, light acts on the energy envelope and these vibrations are transmitted to the cellular processes resulting in an enhancement of the energy that contributes to enhanced physiological function. This in itself has been described as a vibrating system [49].

Using DNA microscopy [103], a change in cellular phase transition can be visualized, which may reflect a change in protein structure that will allow for different functions and perturbations. Phase transitions are not related to chemical functions but to forces and energy parameters within the structure of the cell, including some other thermodynamic properties. The aromatic ring structure of a protein, as indicated by specific paired and un-paired electron distributions, dictates its resonance frequency.

Research into the neural mechanisms of PBMt commonly explores changes in cellular activity [145]. In this context, the electrical energy produced by the action potentials of neurons is of particular importance [146]. However, non-sinusoidal “global” neural oscillations, typically measured by EEG [147], are another form of electrical activity in the brain which are often overlooked. These brain waves are formed by the synchronized activity of individual neurons within a network, creating symphonic wave-type electrical oscillations at a lower frequency than the action potential of a single neuron [148]. Macromolecules resonate at a higher frequency compared to the slower oscillations of brain waves. Currently, there is evidence that PBMt changes the cortical connectome in the same way as ambient light [149,150,151], which is important because the oscillatory networks; theta, beta, alpha, gamma, are widely acknowledged as being important in health and disease [152,153]. In particular, gamma oscillations (25–140 Hz) have been well reviewed [154].

In one study, PBMt at different wavelengths was able to affect the neural oscillations, particularly in the gamma range, by increasing gamma oscillations and improving coherence during task-related activity [93]. Interestingly, oscillations were unchanged at rest after PBMt exposure; however, subsequent studies have shown that modulation of oscillations is also possible during rest [50]. In addition, transcranial PBMt with near-infrared (NIR) light at a wavelength of 810 nm and frequency of 40 Hz changed the default mode network and increased the power of the higher oscillatory frequencies of alpha, beta, gamma [50]. It is likely that this modulation of the default mode network is influenced by neurochemical concentrations of glutamate and gamma aminobutyric acid (GABA), as a high concentration of glutamate in the posterior cingulate cortex and pre-cuneus area is reportedly linked to reduced neural deactivation [155]. Interestingly, cortical spreading depression, which is characterized by increased glutamate and extracellular K^+^ concentration, is one cause of migraine with aura [156], another pathology characterized by abnormal cortical coherence [157]. Additionally, migraine without aura is associated with disrupted default mode network connectivity [17].

The application of different wavelengths, by specific order of application or simultaneous application, can significantly change the oscillation activity in neurons [133]. These changes may be due to the cross-interaction of melanocortinergic and dopaminergic systems resulting in neural modulation [158], which in turn depend on membrane action potential wave-forms in pyramidal neurons of the prefrontal cortex [159,160]. These modulations may have direct implications for the way that PBMt should be used to modulate neural oscillations for the treatment of pathologies that manifest in impaired neural oscillatory networks. This includes an understanding of the types of neural oscillation variation.

### 4.2. Wavelength Specificity and Protein Interactions—Photophysical Resonance

The commonality between laser-produced light and proteins is in their mutual oscillatory properties. Indeed, two entities vibrating at equal natural frequencies can interact with and activate one another. This is part of the so-called resonance phenomenon. Specifically, this model portrays protein-to-protein interactions as occurring via resonant EM energy transfer [29]. This experimentally-validated hypothesis [161] has significant implications for PBMt because the EM resonance is within the range of infra-red and visible light [29]. The spectral and space analysis of a protein can determine the distribution of free energy electrons, which have specific frequencies for specific functional groups [162,163]. 

These principles present the prospect, in precision medicine, of laser intervention to target specific proteins. The inherent resonant properties of proteins are shared with the coherent light produced by lasers. Since the EM resonance energy transfer is integral to protein-to-protein activation, it is expected that the laser wavelengths used for PBMt can interact with the same proteins resonating at equal frequencies. Indeed, this hypothesis accounts for the importance of wavelength in achieving beneficial results in therapy. In particular, signaling proteins have autofluorescence characteristics in the infrared range [161], which may explain why infrared and near-infrared wavelengths of PBMt are most effective for treating neurological performance [164], generalized anxiety disorder [165], and PD [166].

### 4.3. Fluorescent/Auto-Fluorescent Proteins

Beyond the photophysical resonance of endogenous molecules and cells, certain fluorescent/auto-fluorescent molecules will also have an influence, if not interplay, with existing oscillations, or those modulated by PBMt. In this context, cellular auto-fluorescence, as defined by Surre et al. [167] denotes the production of intrinsic natural fluorescence deriving from fluorescent cell structures and metabolites, with common examples including flavins, NAD, aromatic amino acids, lipofuscins, advanced glycation end products, and collagen [167,168,169].

Indeed, it has been shown that DNA molecules can naturally fluoresce once they briefly exit their “dark state”; an extended time period wherein they do not absorb nor emit light, and the fluorescence process can be initiated by applying a certain wavelength of light [100]. It is possible that non-auto-fluorescent proteins, when subjected to the correct excitation wavelength, will also absorb light and initiate downstream processes. Moreover, the implications of fluorescent proteins being specific directional light absorbers and emitters suggests that interventions that utilize light to elicit a physiological response, or in the detection of biological activity, may also be dictated or modulated by the directional properties of proteins. Indeed, targeting cellular autofluorescence for measurements of metabolic activity and other diagnostic investigations has been previously shown and the numbers of tissue and cellular types found to be able to produce autofluorescence is increasing, with studies reporting naturally occurring detectable fluorescence from white blood cells [170], fibroblasts [171], the liver [172,173,174,175], and the kidney [55].

Finally, the connection between endogenous resonance and fluorescence is revealed by linking two separate studies that investigated novel methods to discriminate cancerous cells. The first used biophotonic analysis to demonstrate that stressed, cancerous cells emitted shorter wavelengths of light compared to non-cancerous cells, which emitted photons within the near-infrared range [161]. This demonstrates that the EM nature of a cell is altered according to its health. Equally important is the identification of a resonance fingerprint of cancer DNA, at approximately 1.6 THz, which is a specific signal possibly due to aberrant methylation [176]. Together, these studies highlight the potential for an interactive relationship between endogenous resonance and biophoton emission. Resonant polarity and direction are also currently being investigated to determine the importance of the angle of light emission [177], which further demonstrates the difference between results using laser PBMt with coherent light, compared to LEDs.

## 5. Current Clinical Applications

The clinical applications of the photophysical pathways go hand-in-hand with the phototransductive mechanisms underlying the effects of PBMt in health and disease. Any pathology that involves photosensitive proteins that are responsive to light-induced oscillation modulations, that can induce global oscillatory interactions, could be considered to be amenable to PBMt as a therapeutic option.

### 5.1. Resonance

**Figure 6:**
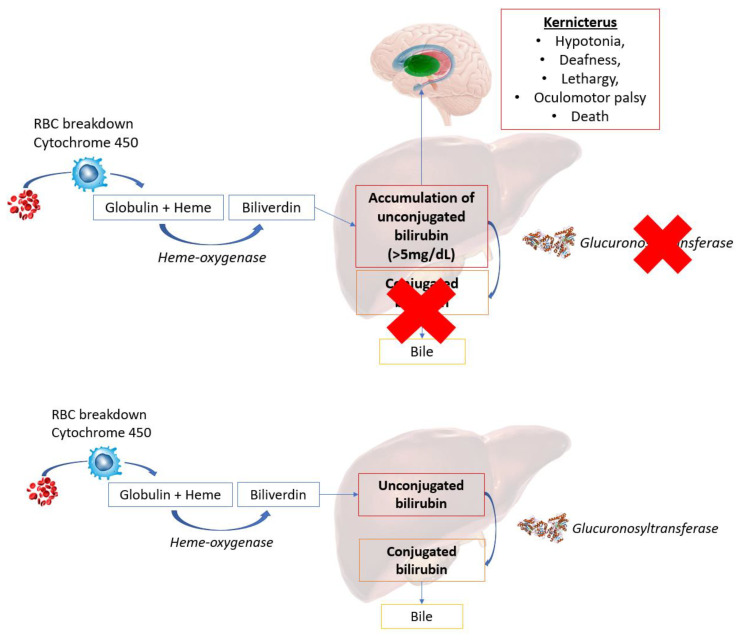
A schematic comparing functional and dysfunctional glucuronidation of unconjugated bilirubin in the liver. Dysfunctional glucuronidation is characteristic of Crigler-Najjar syndrome.

A clinical example of light therapy and its interaction with resonance theory at the protein scale is the treatment of Crigler-Najjar syndrome. This rare syndrome is characterized by a lack of 5′-diphosphglucuronosyltransferase 1-A1 (UDP) activity, which plays a role in glucuronidation of unconjugated bilirubin in the liver [178] (see Figure 6). To date, the most effective treatment of this syndrome in babies and young people is administering blue light phototherapy to the patient [43].

The effectiveness of blue light phototherapy for Crigler-Najjar syndrome may be due to the EM modulation of cellular proteins. Experimentally, the function of human protein UDP in bilirubin metabolism has been analyzed using the resonant recognition model (RRM), which showed characteristic frequencies associated with the blue light spectrum [43]. This presents a biophysical relationship between the UDP protein characteristic frequency and the wavelength of blue light. It is therefore proposed that blue light may serve as an “imitator” of the resonant activity typically present in UDP [43].

There may be other diseases besides Crigler-Najjar that can be managed in a similar way, including illnesses characterized by abnormal neural oscillatory activity (especially with reference to changes in the microtubular and cytoskeletal network) and those with pathologies in the central and peripheral nervous systems, such as PD, AD, chronic pain and inflammation, autism, and migraines [18,157,179,180,181,182,183,184,185,186].

### 5.2. Neutrophils

Practical applications of modulation of neutrophil processes are important in the treatment of inflammatory lung disease and other diseases of inflammation [187]. Mechanotransduction plays a key role in neutrophil activation and deactivation [188]. An understanding of the photophysical aspects of mechanotransduction activation in neutrophils will be crucial in precision medicine for conditions with motor dysfunctions, such as neurodegenerative conditions and cardiac diseases.

PBMt has a considerable effect on neutrophil function particularly in increasing neutrophil phagocytosis efficiency and in modulating the concentration of neutrophils produced during an immune response, particularly in the lungs [68,187]. This may be important in the observed decrease of neutrophils with aging [189], the impairment of neutrophils in cardiac diseases [190,191], PD [192,193,194,195] and increasingly in viral load sequelae, including the current COVID-19 pandemic [196]. A recent review supported PBMt as an adjunctive treatment of lung inflammation and for rehabilitating other affected organs by modulating neutrophil influx and inhibiting the macrophage inflammatory protein-2 and thereby reducing pulmonary edema [197].

It can be postulated that dysfunctional non-visual phototransduction processes, including photonic production, may also reflect disease processes involving neutrophils, including in the heart, since neutrophil membranes are a major source of photonic production [198,199]. This is particularly important in diseases concerning DNA aberrant methylation, such as chronic pain [200,201] and cancer [202,203,204]. Additionally, there are several diseases that involve abnormal neutrophil activation [205,206,207], for example, an abnormal lung response to air-borne toxins such as those found in air pollution, causing the dysfunctional activation of neutrophils [208,209], which may be modulated by PBMt. There is evidence that PBMt can modulate neutrophil activation, both by decreasing excessive neutrophil response and making the neutrophil burst more effective [197,210]. There is also increasing evidence that physiological processes have a biophotonic emission signature [53] that is different from the signature of pathophysiological processes [161,211,212].

### 5.3. Channelopathies

Mechanotransduction plays an essential role in myocardial mechano- and electro-physiologic function. It involves an assembly of protein complexes to mediate the sensing and transmission of mechanical or electrical loads. These proteins, largely within the sarcomere, intercalated disc, and sarcolemma of myocardial cytoskeleton, trigger cascading intra- and inter-cellular processes, possibly effecting anatomic and physiologic or pathophysiologic alternations [213].

Genetic mutations in intracellular processes of mechanotransduction are the responsible agents in some of the channelopathies and cardiomyopathies, such as arrhythmias, including sudden death and heart failure [214,215]. Importantly, the disruption of mechanotransduction pathways has also been shown to play a significant role in initiation, and the progression, of other cardiovascular diseases including atherogenesis, hypertension, and atrial fibrillation [216,217].

PBMt may offer novel therapeutic applications in relation to mechanotransduction anomalies of the heart. PBMt has been recently shown in experimental models to resolve atrial fibrillation [218] and cardiac pacing utilizing an optogenetics approach [219]. Additionally, a recent study revealed that an optogenetic approach can also be used to activate dopaminergic neurons of the substantia nigra pars compacta in an experimental animal model [220]. This was achieved through upregulating levodopa (L-DOPA) production by recovery of tyrosine hydroxylase, which has implications for PBMt.

### 5.4. Analgesia and Anaesthetic Effects

The analgesic properties of PBMt, beyond the resolution of cortical coherence and brain wave pattern disruptions, are supported by a wealth of data that provide insight into the possible delivery of pre-emptive PBMt in the prevention and development of persistent pain [221], including neuropathic pain [87] chemotherapies [222], neck pain [223], low back pain [224], and pain following nerve or spinal cord injury [225]. Hypothetically, reversible cytoskeletal disruption may modulate pain by disruption of cytoskeletal and microtubular structures to physically interrupt ATP delivery and block neuronal depolarization to limit afferent signaling to the dorsal horn and through the disruption of fast axonal flow and limiting the transport of pro-inflammatory cytokines, as is evident via the appearance of dendritic varicosities.

A recent review evaluated the potential role of transcranial PBM as an adjuvant to enhance the effects of pharmacological anesthetics, coining the term “Optianesthesia” to describe this effect [226]. In this context, transcranial PBM at wavelengths of 808 nm or 810 nm had inhibitory effects on the cortex and hippocampus of healthy rats [227], showing possible therapeutic effects with reported attenuation of pharmacologically-induced seizures [228,229,230]. These observations were also reported when transcranial PBM was combined with valproic acid in the same model [231]. Furthermore, application of transcranial PBM has been shown to elicit anticonvulsant effects, showing evidence of abnormal electrical discharge inhibition. Based on these reports, it could be speculated that PBM delivered transcranially may be a promising adjuvant or add-on therapy in combination with general anesthesia to treat pediatric refractory status epilepticus and super refractory status epilepticus, which may in turn reduce some of the side effects experienced following administration of anesthetics [226]. To achieve this, it is possible that PBM may be altering consciousness reversibly via modification to quantum processes in microtubules that underly consciousness, in a similar way that general anesthetics can bind and affect microtubules to influence consciousness [232,233,234], including acting on quantum ion channels in neuronal microtubules specifically in brain regions known to be targeted by general anesthetics [57,232,235,236]. It is also suggested that transcranial PBM may aid in the distribution of pharmacological general anesthetics. There is evidence to suggest that PBM application has an arousal-dependent effect, that when applied during wakefulness is able to stimulate neuronal functions, such as increased mitochondrial activity and gene expression, as well as influence alpha, beta, and gamma waves and enhance neuronal protection and survival against distress and neurodegenerative diseases [60]. When PBM is applied during sleep, it is possible that there may be increased clearance of cerebral spinal fluid, which may be due to an increase in the permeability of AQP4 in astrocytes [60]. These mechanisms may be relevant when the maintenance phases induced by using intravenous and inhaled anesthetics are considered, whereby the PBM-induced increase in cerebral spinal fluid flow in tandem with general anesthetic administration may have a synergistic effect in their speed of delivery and eventual distribution in the body.

### 5.5. Wounds and Aging

Increasing numbers of studies have reported evidence of downstream epigenetic changes following PBMt application, including changes to histone acetylation and DNA methylation that have consequential effects on functional cell maturation [237]. For example, PBMt for epithelial wound healing has shown accelerated epithelial migration and chromatic relaxation, along with increased levels of histone acetylation and the expression of cyclic AMP response element-binding protein (CBP) p300 and the mammalian target of rapamycin (mTOR) [238]. PBM was also shown to reduce levels of the transcription repression-associated protein methyl-CpG-binding domain proteins (MBD2), along with decreased numbers of epithelial stem cells and spheres [238], making it plausible that PBMt can induce epigenetic changes to epithelial cells to accelerate healing. Similarly, transcranial PBM was reported to increase signaling proteins related to both cell proliferation and cell survival [239]. Similar changes were also reported underlying oral ulcer repair using PBMt, which accelerated repair of oral ulcers and increased both histone 3 acetylation and NF-kB positive cells [240]. Interestingly, prolonged PBM application resulted in a reduction of histone 3 acetylation and NF-kB cells, suggesting that PBMt can stimulate keratinocyte migration during the initial phases of epithelial wound healing, followed by keratinocyte differentiation during the final stages [240]. Signal transducer and activator of transcription 3 (STAT3), extracellular signal-regulated protein kinase (ERK), c-Jun N-terminal kinase (JNK), p70 ribosomal protein S6 kinase (p70S6K) and protein kinase B (PJB) were all shown to be modulated following PBM application in a rat model. PBM was shown to activate STAT3, ERK and JNK signaling proteins in the cerebral cortex, while the increased expression of p70S6K and STAT3 and the activation of Akt were observed in the hippocampus. PBM was also shown to improve intracellular signaling pathways linked to cell survival, memory and glucose metabolism in a aging rat brain model [239].

## 6. PBMt and Neuro Oscillatory Networks: Clinical Implications

The ability to modulate impairments in neural oscillatory networks is especially relevant to PD [183,186], where neural gamma oscillations are impaired in individuals with the disease. Gamma oscillations are vital in cortico-basal ganglia loops which govern motor control [19], and thus their dysfunction gives rise to typical Parkinsonism symptoms such as resting tremors. Now that it has been shown that gamma oscillations can be modulated with transcranial PBMt [50], clinicians may be able to better manage the symptoms of PD. The early results of one ongoing study on PD patients using LED-lined cranial “buckets” have suggested an overall improvement in symptoms in more than half of the participants [241], although high-quality trials with larger participant numbers are needed.

Migraine is partly characterized by abnormal cortical coherence [179,182] and is also defined as a channelopathy [242], which is an impairment of ion channels and their receptors [243], and in turn shares a common etiology with epilepsy [244]. It has been postulated that genetic channelopathies have implications in chronic pain conditions, as well as acquired channelopathies such as from trauma and whiplash [245,246]. Migraine is one neurological condition where targeting neural oscillatory irregularities may be a plausible form of treatment. Migraineurs present with an abnormally increased amplitude of low-frequency oscillations (LFOs) in the thalamocortical networks [157]. The increased amplitude of these LFOs, which are characterized as delta oscillations, positively correlates with the increased frequency of headaches. Therefore, the defective thalamocortical brain-wave activity of migraineurs may predispose migraineurs to repeated episodes [157] and could therefore be a therapeutic target for the suppression of subsequent migraines. Transcranial PBMt has been shown to reduce the power of delta oscillations [50], and the rhythmic cortical feedback to the thalamus influences thalamic oscillatory behaviors [157]. Thus, light that penetrates through the scalp and reaches the cortex may modulate low frequency oscillations in sub-cortical structures. Since it is now known that structures such as the suprachiasmatic nuclei in the hypothalamus show tissue-level rhythms during fetal development, before clock gene expression introduction [247], it is plausible to postulate that external triggers, such as PBM irradiation, could elicit a response from environmental factors affecting innate oscillation.

Gamma oscillations are also impaired in individuals with widespread centralized pain in fibromyalgia [18], schizophrenia, cognitive disorders, and other neurodegenerative diseases, including AD [180,181,185]. Autism has also been shown to involve impairment of cortical coherence [184] and would also be a potentially novel target for treatment with PBMt [248]. Interestingly, AD is of particular relevance due to its characteristic pathology of fibrillated amyloid-β. Indeed, amyloids typically have optical absorption properties, yet this feature is absent in the fibrillated state [37]. Other studies have demonstrated effects of PBMt on sleep enhancement [249], which is often reported as a positive side-effect of transcranial PBMt [165,250,251]. The fact that the oligomer length of amyloid-β bi-directionally modifies sleep [252], combined with the optical absorption properties of amyloid-β [37], presents the possibility that amyloid-β may be directly affecting sleep in patients and is a target for patients receiving PBMt. Further, neural oscillations are associated with quality of sleep and are vital to achieving deep stages of sleep (rapid eye-movement (REM) sleep) [253], suggesting that the observed effects may be explained by a neural oscillation modulation mechanism.

There is clear evidence that transcranial PBMt modulates cortical oscillatory behavior, however to date, there is a debate as to the mechanism involved. It is proposed that propagating biophotonic and electrical signaling may become synchronized so as to alter the slow waves in the body, including the brain. When groups of neurons synchronously “fire” action potentials, they can form larger, slower waves in the brain [148]. It is therefore possible that the high frequency oscillations of DNA may interact on a “local” scale with proximal DNA from other cells, to combine and produce global, slower forms of oscillation in the brain and body. Another possible mechanism by which PBMt affects global circuits is through interactions with other novel oscillations, such as calcium, mitochondrial, or astrocyte oscillations [254,255,256].

## 7. Future Implications of Photophysical PBMt Mechanisms Applied to Clinical Therapy

The implications of this photophysical hypothesis become significant when the development of treatment and management of diseases that are characterized by abnormal neural oscillations, such as migraine, PD, autism, and AD are considered. Since neural oscillations can be modulated by transcranial PBMt [50,93,257,258], this intervention may be an effective therapeutic application for these pathologies. The evidence of a modulatory mechanism in healthy participants is promising [93,94,259], although further studies are needed to evaluate the effect of PBMt treatment on symptomatology.

The RRM can be used in designing the formation of novel antimicrobial peptides in the treatment of skin cancers [260] and pathologies involving dysfunctions in neurogenesis [261,262]. Promoters or inhibitors designed using the RRM can modulate the quantity of biphoton emissions to either facilitate or block release. One study has shown that malignant cells emit blue wavelengths of light compared to healthy cells, which emit mainly biophotons in the infrared bandwidth [161]. Additionally, a recent study reported biophoton analysis to be useful in the discrimination of precancerous cells [161]. This non-invasive method utilizes wavelength-exclusion filters on cell cultures to determine the wavelength and amount of biophoton emission. By extension, this may have significant implications for the early detection of disease since the wavelength of the emitted biophotons appears to be indicative of cellular health. In summary, stressed cells may be subject to phase changes that influence the wavelength of light that they produce.

## 8. PBMt and Precision Medicine

The photophysical mechanisms described here combine known biological and biophysical phenomena to explain novel cellular signaling and their associated downstream effects [263]. The hypotheses presented here are multidisciplinary and comprises mechanisms beyond cytochrome c oxidase activation and offer a new perspective on the photophysical effects of PBMt and its relevance in the optimization of health outcomes and performance.

The potential signaling pathways employed during cell-to-cell communication utilizing biophotons [47] presents the possibility that externally applied light via PBMt may modulate these pathways and instigate cellular processes such as protein conformational changes in PBMt treated cells [38]. The RRM concept [162] underpins the local oscillatory protein signatures and forms the basis of our proposed perspective on the similarities between micro-oscillations observed following either PBM light-induced interactions with proteins or activation by ATP. The wavelength at which proteins oscillate influenced by endogenous cell biophotons, would inform on precision medicine. In the case of laser PBM, the light is coherent and oscillates at a specific frequency depending on the wavelength and thus can interact (according to RRM) with proteins resonating at equivalent frequencies. This is illustrated clinically through the blue light treatment of Crigler-Najjar syndrome, with the wavelength of light sharing a comparable resonant frequency with the UDP protein.

These local resonant interactions may also explain recent evidence for neural oscillation modulation by transcranial PBMt [50], which may be important in treating pathologies associated with the impairment of neural oscillations such as pain in fibromyalgia [18], schizophrenia, cognitive disorders, autism [184], migraine, and neurodegenerative diseases, such as PD and AD [180,181,185]. Beyond electrical signaling, there exists neurotrophic signaling and fast axonal flow, protein-oscillation communication, and electric oscillations of microtubules [264,265,266]. This latter mechanism may involve microtubule dynamics in the regulation of excitability in neurons, and involve the presence of ion channels, including potassium channels such as TRESK [45]. Clinically, this is important in migraine with aura and related diseases that involve this mutation, a well as other dysautonomia-related diseases, including PD and cardiac disease.

Future experimental and clinical studies on PBMt should determine the precise wavelengths efficacious for specific disease processes. Biophotonic analysis of the brain should be investigated during transcranial PBMt so as to determine if there is an increase in biophoton release and which wavelengths are being emitted.

## 9. Conclusions

PBM affects a wide range of biologic and pathologic processes. Our paper adds new pieces of information to the existing literature on PBM’s complex mechanisms of action as modulators of cellular function and metabolic pathways as well as introducing host/microbiota interactions in health and disease. Knowledge of photophysical mechanisms would be beneficial in guiding the future design of experimental and clinical studies of PBMt. This could include synergistic transcranial and systemic applications of PBMt. The knowledge of photophysical mechanisms, especially oscillatory and resonance modulation mechanisms, could therefore be utilized to identify treatment outcomes with different precision applications of PBMt and foster future analytical studies of varying biophotonic activity at the tissue and molecular level. By considering the whole body as a system with interacting oscillating components, light allows an enhancement of the energy that makes the physiological processes tend towards better function. This may have implications for the vibrating body system as a whole and the loss of this vibration in aging. The potential mechanisms of PBM beyond cytochrome c oxidase presented here may well overlap in their modes of action, producing synergistic or complementary outcomes.

The perspectives presented here might also offer a new insight and drive future research into the global photophysical effects in PBMt, which may be important when considering the relevance of PBMt in clinical applications, including the treatment of diseases, especially inflammatory diseases of various neurological and metabolic disorders with a focus on optimization of health outcomes and performance.

PBMt as a non-invasive, low-risk modality can deliver precision medicine for various diseases. The therapeutic regimen of light therapy and mode of delivery can then be individualized to target specific disease processes utilizing the most effective mechanistic pathways.

## Figures and Tables

**Table 1:** Seminal studies informing on changes in the light therapy landscape.

Authors	Title	Contribution
Gurwitsch 1932 [23]	Mitogenetic Emission	Release of biophotons
Popp et al., 1984 [24]	Biophoton emission. New evidence for coherence and DNA as source	Release of biophotons
Kert & Rose 1989 [25]	Low level laser therapy	Clinical applications of PBM
Albrecht-Buehler 1992 [26]	Rudimentary form of cellular “vision”	Release of biophotons
Laakso et al., 1993 [27]	Quality of light—is laser necessary for effective photobiostimulation?	PBM coherence
Amano et al., 1995 [28]	Ultraweak biophoton emission imaging of transplanted bladder cancer	Biophotons for diagnosis
Cosic 2001 [29]	The Resonant Recognition Model of Bio-molecular Interactions: possibility of electromagnetic resonance	Resonant oscillation theory
Voeikov et al., 2003 [30]	Biophoton research in blood reveals its holistic properties	Release of biophotons
Amat et al., 2006 [31]	The electric field induced by light can explain cellular responses to electromagnetic energy: A hypothesis of mechanism	PBM coherence
Chow et al., 2007 [32]	830 nm laser irradiation induces varicosity formation, reduces mitochondrial membrane potential and blocks fast axonal flow in small and medium diameter rat dorsal root dorsal root ganglion: implications for the analgesic effects of 830 nm laser.	PBM modulation of cytoskeleton
Mathew et al., 2010 [33]	Signalling effect of NIR pulsed lasers on axonal growth	Biophoton signaling
Sun et al., 2010 [34]	Biophotons as neural communication signals demonstrated by in situ biophoton autography.	Communication with biophotons
Bokkon et al., 2010 [35]	Estimation of the number of biophotons involved in the visual perception of a single-object image: Biophoton intensity can be considerably higher inside cells than outside	Communication with biophotons from periphery to brain
Minke 2010 [36]	The history of the Drosophila TRP channel: the birth of a new channel superfamily	Photon activation of neuronal ion channels
Lavi et al., 2012 [11]	The Plasma Membrane is Involved in the Visible Light–Tissue Interaction	PBM membrane interactions
Hanczyc et al., 2013 [37]	Multiphoton absorption in amyloid protein fibres	Photons for diagnosis
Liebert et al., 2014 [38]	Protein conformational modulation by photons: A mechanism for laser treatment effects	Biophoton theory for PBM
Niggli 2014 [39]	Biophotons: ultraweak light impulses regulate life processes in aging	Biophotons for diagnosis
Tang & Dai 2014 [40]	Spatiotemporal imaging of glutamate-induced biophotonic activities and transmission in neural circuits	Communication with biophotons in the brain
Budagovsky et al., 2015 [41]	Cell response to quasi-monochromatic light with different coherence	Oscillation theory
Shi et al., 2016 [42]	Photon entanglement through brain tissue	Quantum entanglement theory
Cosic & Cosic 2016 [43]	The treatment of Crigler-Najjar syndrome by blue light as explained by resonant recognition model	Clinical application of resonance theory
Poznanski et al., 2017 [44]	Solitonic conduction of electrotonic signals in neuronal branchlets with polarized microstructure	Soliton and nerve theory
Cantero et al., 2018 [45]	Bundles of brain microtubules generate electrical oscillations	Photon modification of microtubules in neurons
Johnson & Winlow 2018 [46]	The Soliton and the Action Potential—Primary Elements Underlying Sentience	Soliton and nerve theory
Fekrazad 2018 [47]	Photons Harmony for Cell Communication	Biophotons and PBM
Santana-Blank & Rodríguez-Santana [48]	Photobiomodulation in Light Our Biological Clock’s Inner Workings	PBM and circadian oscillations
Facchin et al., 2019 [49]	Physical energies to the rescue of damaged tissues	Biophotons and PBM
Zomorrodi et al., 2019 [50]	Pulsed near infrared transcranial and intranasal photobiomodulation significantly modulates neural oscillations: a pilot exploratory study	PBM and neural oscillations
Wang et al., 2019 [51]	Transcranial photobiomodulation with 1064-nm laser modulates brain electroencephalogram rhythms	PBM and neural oscillations
Lima et al., 2019 [6]	Photobiomodulation enhancement of cell proliferation at 660 nm does not require cytochrome c oxidase	PBM photophysical mechanisms
Pope et al., 2020 [52]	Wavelength-and irradiance-dependent changes in intracellular nitric oxide level	PBM photophysical mechanisms
Esmaeilpour et al., 2020 [53]	An Experimental Investigation of Ultraweak Photon Emission from Adult Murine Neural Stem Cells	Biophotons for diagnosis
Sordillo & Sordillo 2020 [54]	The mystery of chemotherapy brain: kynurenines, tubulin and biophoton release	Clinical application of biophotons
Mahbub et al., 2020 [55]	Non-invasive real-time imaging of reactive oxygen species (ROS) using auto-fluorescence multispectral imaging technique: A novel tool for redox biology	Autofluorescence
Zangari et al., 2021 [56]	Photons detected in the active nerve by photographic technique	Biophotons and nerve theory
Staelens et al., 2022 [57]	Near-Infrared Photobiomodulation of Living Cells, Tubulin, and Microtubules	Photon modification of microtubules in neurons
Korneev et al., 2022 [58]	Exploring Structural Flexibility and Stability of α-Synuclein by the Landau–Ginzburg–Wilson Approach	Solitons
Moro et al., 2022 [59]	The code of light: do neurons generate light to communicate and repair?	Biophoton communication
Moro et al., 2022 [60]	The effect of photobiomodulation on the brain during wakefulness and sleep	Biophoton and circadian rhythms

## Data Availability

No new data was created.

## Abbreviations

AD	Alzheimer’s disease
AQP	Aquaporin
ATP	Adenosine triphosphate
PBMt	Photobiomodulation therapy
CASP-9	Caspase-9
CSF	Cerebrospinal fluid
DRG	Dorsal root gangion
EM	Elctromagnetic
GABA	gamma aminobutyric acid
H_2_O_2_	Hydrogen peroxide
JAK-STAT	Janus kinases-signal transducer and activator of transcription proteins
KHz	Kilohertz
L-DOPA	Levodopa
LED	Light-emitting diode
LFOs	Low-frequency oscillations
MAP2	Microtubule-associated protein 2
MMP	Mitochondrial membrane potential
NIR	Near-infrared
NO	nitric oxide
PD	Parkinson’s disease
REM	Rapid eye-movement
ROS	Reactive oxygen species
RRM	Resonant recognition model
SPD	Spectral power density
THz	Terahertz
TRESK	TWIK-related spinal cord potassium channel
TRP	Transient receptor potential
TWIK	the weakly inward rectifying K channel
UDP	5′-diphosphglucuronosyltransferase 1-A1
UPE	Ultra-weak photon emissions
VE	vascular endothelial
